# Exploratory evaluation of spinal cord stimulation with dynamic pulse patterns: a promising approach to improve stimulation sensation, coverage of pain areas, and expected pain relief

**DOI:** 10.3389/fpain.2023.1339892

**Published:** 2024-01-31

**Authors:** Changfang Zhu, Rosana Esteller, Jessica Block, Kristen Lechleiter, Robert Frey, Michael A. Moffitt

**Affiliations:** ^1^ Research and Development, Boston Scientific Neuromodulation, Valencia, CA, United States; ^2^ Clinical Research, Boston Scientific Neuromodulation, Valencia, CA, United States; ^3^ Pacific Pain Management Inc., Ventura, CA, United States

**Keywords:** neuromodulation, dynamic, pattern, paresthesia coverage, sensation and perception, pain relief, pulse sequence

## Abstract

**Background:**

The societal burden of chronic pain and the contribution-in-part to the opioid crisis, is a strong motivation to improve and expand non-addictive treatments, including spinal cord stimulation (SCS). For several decades standard SCS has consisted in delivery of tonic pulses with static parameter settings in frequency, pulse width, and amplitude. These static parameters have limited ability to personalize the quality of paresthesia, the dermatomal coverage, and thus may affect SCS efficacy. Further, static settings may contribute to the build-up of tolerance or loss of efficacy of the therapy over time in some patients.

**Methods:**

We conducted an acute exploratory study to evaluate the effects of SCS using time-dynamic pulses as compared to time-static (conventional tonic) stimulation pulses, with the hypotheses that dynamic pulse SCS may enable beneficial tailoring of the sensation and the patient's expectation for better pain relief with SCS. During a single clinic visit, consented subjects undergoing a standard SCS trial had their implanted leads temporarily connected to an investigational external stimulator capable of delivering time-static and six categories of time-dynamic pulse sequences, each characterized by continuously varying a stimulation parameter. Study subjects provided several assessments while blinded to the stimulation pattern, including: drawing of paresthesia maps, descriptions of sensation, and ratings for comfort and helpfulness to pain relief.

**Results:**

Even without optimization of the field location, a majority of subjects rated sensations from dynamic stimulation as better or equal to that of static stimulation for comfortableness and for helpfulness to pain relief. The initial data showed a gender and/or pain dermatomal location related preference to a stimulation pattern. In particular, female subjects and subjects with pain at higher dermatomes tended to rank the sensation from dynamic stimulation better. Dynamic stimulation produced greater pain coverage without optimization; in 70% (9/13) of subjects, maximal pain coverage was achieved with a dynamic stimulation pattern. There was also greater variety in the words used by patients to describe stimulation sensation in the free text and free form verbal descriptions associated with dynamic stimulation.

**Conclusions:**

With the same electrode configuration and comparable parameter settings, acute SCS using dynamic pulses produced more positive ratings, expanded paresthesia coverage, and greater variation in sensation as compared to SCS using static pulses, suggesting that dynamic stimulation has the potential to improve capabilities of SCS for the treatment of chronic pain. Further study is warranted.

**Trial Registration:**

This study was registered at ClinicalTrials.gov under ID NCT02988713, November 2016 (URL: https://clinicaltrials.gov/ct2/show/NCT02988713).

## 1 Introduction

Chronic pain is one of the most prevalent disabling health conditions among adults all over the world (1). In 2019, 20.4% of adults in the United States were estimated to have chronic pain (2, 3), which has been associated with substantial limitations in regular living activities (4) and decreased quality of life (5). Prevailing treatment options for this condition often lead to opioid dependence (6, 7), drug overuse (8–10), and poor mental health (5, 11, 12).

Spinal cord stimulation (SCS) has been used as a nonpharmaceutical therapy for treating mixed types of chronic and neuropathic pain conditions (13–20) since its first use for pain treatment in the late 1960 s when Shealy et al. implanted the first spinal cord stimulation system in a terminal cancer patient (21). Their pilot work was inspired by the “gate control theory” (22, 23) which postulated that activation of large myelinated afferents inhibits nociceptive transmission in the spinal dorsal horn. However, the mechanism of action of SCS and how the electrical stimulation delivered to the spinal cord modulate the neuronal activity and perception along the pain processing pathway is complicated and far from fully understood (13, 17, 24).

For decades, the use of SCS therapy has largely been limited to the delivery of continuous tonic or intermittent burst (25, 26) trains of electrical pulses with time-invariant pulse parameters, effectively providing time-static stimulation. These time-static pulses are applied through an epidural or paddle electrode array (lead) chronically implanted over the dorsal spinal cord and connected to an implantable pulse generator. The main static parameters defining these pulses are current amplitude, frequency or rate, and pulse width. In the case of burst stimulation, although the timing of the pulses is defined with two frequency parameters, known as the intra-burst frequency and the inter-burst frequency that result in intermittent bursts of pulses, the two frequencies are still static parameters as they do not change once defined.

Chronic pain patients using standard static SCS therapies with stimulation settings above perception threshold experience a paresthesia sensation produced by the stimulation, and the prevailing dogma is that covering the pain area with the paresthesia will maximize pain relief. The sensation of paresthesia which is often described as a “tingling” or “buzzing”, is usually well-tolerated by most patients, and the patients can adjust the intensity with their remote-control as desired. For some patients and/or some pain areas, the paresthesia from tonic stimulation is not desirable.

An alternative SCS modality, known as paresthesia-free SCS, uses parameter settings below the patient's perception level, which does not evoke paresthesia, and when done properly can still provide strong pain relief after a wash-in period. Contemporary paresthesia-free therapies are also characterized by static pulses, typically at high frequencies (500 Hz or greater up to 10 kHz) (27–29) or burst stimulation (25, 26). More recently, profound and fast (within minutes) pain relief was achieved using a sub-perception method at low frequencies (15). Although helpful, it is noteworthy that all recent advances in SCS are still deployed through static time-invariant pulse trains, and the optimization of stimulation has been focused on identifying the best constant parameter settings of the pulses for stimulation.

Paresthesia has been commonly viewed as a side-effect accompanying SCS therapy, and little attention has been given to the improvement of paresthesia sensation, other than avoiding it at all together (30). But even in intended paresthesia-free SCS, unpleasant or uncomfortable sensation has been reported when stimulation levels went above perception at times, for example, during postural change (31). And, preference for paresthesia vs. paresthesia-free therapy can vary across patients and change over time (18, 31). Some patients even prefer to feel the paresthesia, which may provide a psychological assurance of being treated (32) or contribute to pain relief through higher threshold neural pathways. Whereas most of patients respond to both therapy modalities (paresthesia and paresthesia-free), about 22% to 25% of patients may only respond to one of these modalities (16, 18, 32). If given the choice, a large portion of patients prefer to keep both therapeutic options, paresthesia-based and paresthesia-free, for the management of their pain (16), therefore effort to improvement should be dedicated to both modalities. In this work, we focused on evaluating the sensation of paresthesia using time-dynamic pulses, appreciating that time-dynamic paresthesia-free stimulation is also a topic of research interest.

The paresthesia sensation from standard SCS is often described as a “tingling” or “buzzing” with limited variation in sensation description, largely due to the static (time-invariant) nature of the stimulation pulses (whereas both peripheral and central neurons often exhibit natural neural spiking that is dynamic and time-varying). Peripheral stimulation for a prosthetic neural interface using time varying dynamic pulses has been shown to induce sensation that was perceived as more natural than the “tingling” paresthesia (33, 34). Deep brain stimulation using temporally non-regular pulses has been shown to improve outcomes of neuromodulation treatment of movement disorders such as Parkinson's Disease (35). A temporally patterned stimulation developed using a neural network model of the dorsal horn, was described as potentially more effective than conventional tonic stimulation for pain therapy (36). A clinical study of acute SCS has shown that an intensity-modulated stimulation produced a similar degree of pain relief as compared to conventional SCS for patients with post-laminectomy syndrome (34). In a preclinical (rodent) evaluation, the data of Edhi et al. (37) suggest that SCS with dynamically varying pulses may improve analgesia, the durability of pain relief beyond stimulation-on periods, and potentially enhance the SCS therapy.

We designed and conducted an exploratory clinical study to evaluate the hypothesis that SCS using dynamic stimulation (time-varying pulse trains) may enable tailoring of paresthesia sensation and pain-paresthesia coverage in patients, and to gain insight into the potential value of dynamic stimulation to improve SCS outcomes.

## 2 Methods

### 2.1 Study design

The study was designed as a prospective, multi-center, non-randomized, single-arm, exploratory study, and reviewed and approved by Western Institutional Review Board (WIRB) [WIRB, now WCG IRB]. Study participants were recruited from patients in a pain management practice who were eligible to receive an SCS screening trial to treat their chronic pain condition. Patients that met any contraindication defined in DFU or were diagnosed with cognitive impairment or exhibited any characteristic that would limit the study candidate's ability to assess pain relief or complete study assessments were excluded. Written informed consent was obtained from each participant prior to enrollment into the study.

Each participant was undergoing an SCS screening trial with a commercially approved SCS system [Precision Spectra SC-1132, Boston Scientific Neuromodulation (BSN)] per directions for use (DFU). Two SCS leads (SC- 2316, BSN) were placed in the thoracolumbar epidural space and were temporarily secured for non-surgical removal at the termination of the trial period, per DFU.

Participants were screened and consented up to 14 days prior to the programming visit, which was typically scheduled at the end of their trial period on the day when the trial SCS leads would be removed. The duration of programming visit varied (0.5–2.5 h) depending on the availability of study participants, and participants could request to conclude the test at any point. At the completion of this single programming visit, the subjects concluded their participation in the study.

### 2.2 SCS stimulation

**Figure 1:**
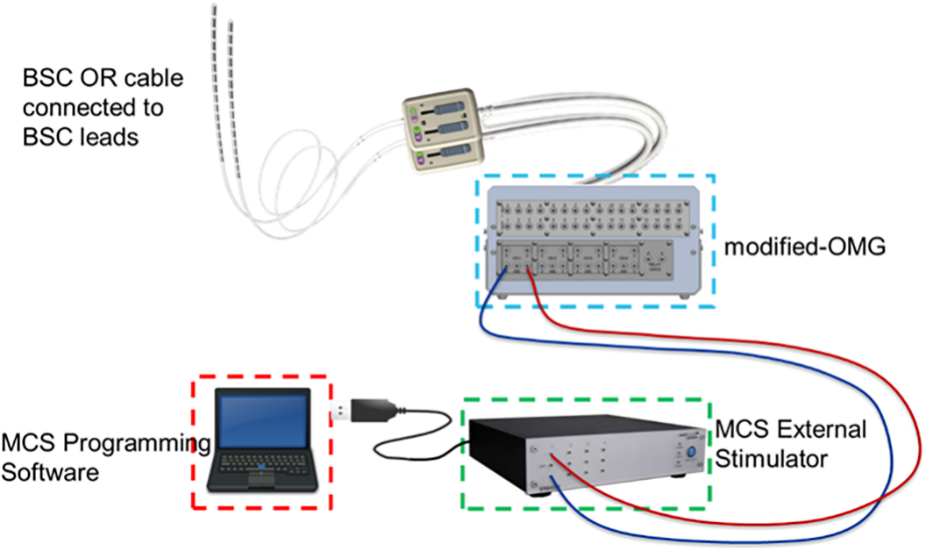
Investigational device system.

Acute SCS stimulation was delivered thorough an investigational device system specifically configured for this exploratory study (Figure 1) which consisted of the Multi-Channel Stimulator (MCS, STG4004, Multi-Channel Systems, Germany), a computer with the MCS programming software (MC Stimulus II), and a modified Observational Mechanical Gateway (m-OMG) designed in-house to provide an interface between MCS and stimulation leads, which included the external OR (operating room) cables and the implanted epidural leads.

**Figure 2:**
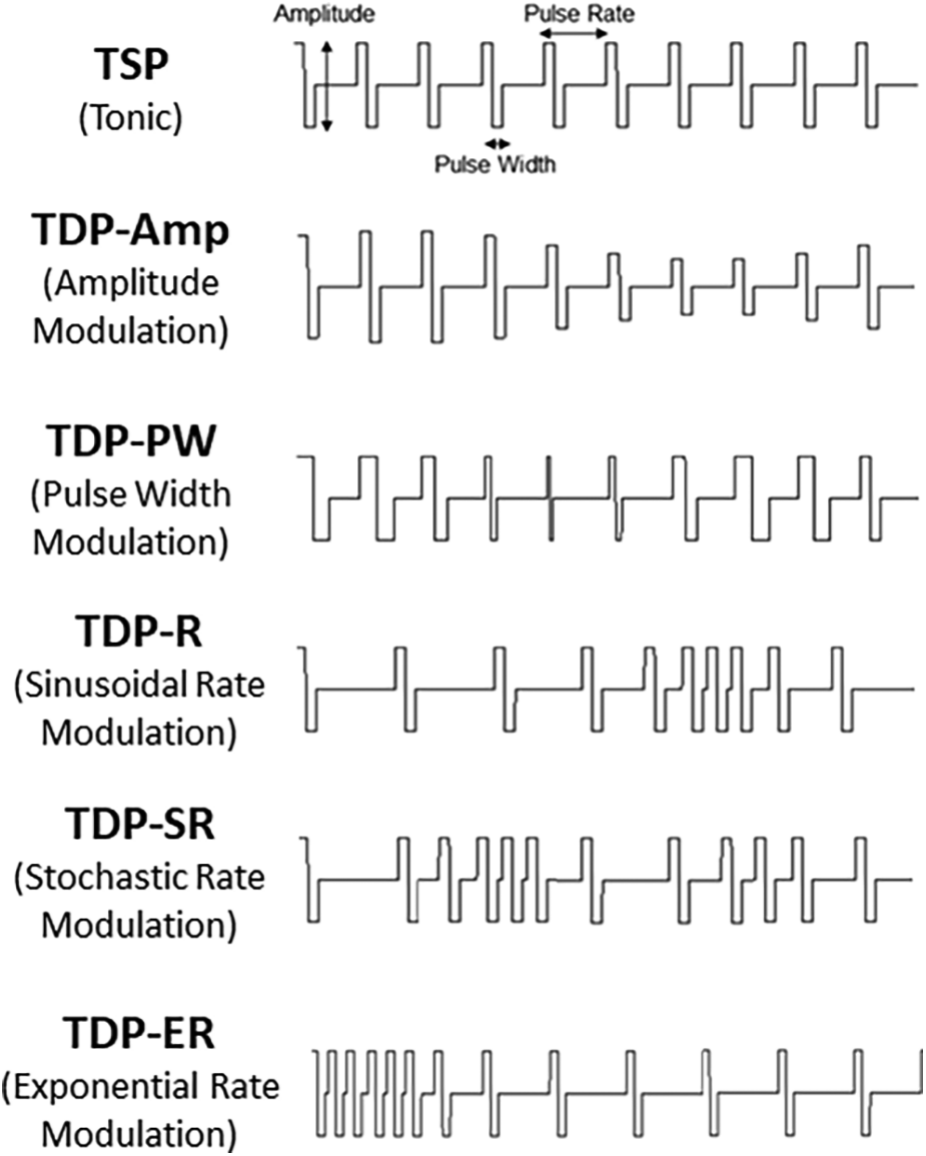
Illustration of the TSP and TDP.

Stimulation pulse sequences consisted of a sequence of pulses with each pulse defined by an amplitude, a pulse width and time until the next pulse (period). Two main types of stimulation sequences were evaluated in the study: time-static pulse (TSP) sequences and time-dynamic pulse (TDP) sequences. TSP stimulation, which used conventional tonic sequences, was delivered in two different ways: one programmed and delivered using the BSN commercial external trial system (ETS) and denoted as TSP-ETS, and the other programmed and delivered through the investigational MCS device and denoted as TSP-MCS. TDP stimulation was delivered by the investigational MCS device and included six categories of time-dynamic pulse sequences where at least one parameter of amplitude, pulse width, or period / frequency (rate) was modulated over time according to a specified function or model. When the modulated parameter was the amplitude or pulse width the pattern was denoted as TDP-Amp and TDP-PW, respectively. In these cases, the pulse width or amplitude was varied over time by adding an increment that varied over time following a sinusoidal function [D% · sin(f_0_t)] while the other parameters were held constant, where D% represents the modulation depth ranging within 10%–100%, and f_0_ represents the modulation frequency ranging within 0.5–2 Hz. For rate modulation sequences, the instantaneous pulse rate (frequency) was modulated by adding an increment that varied over time following a sinusoidal function [sin(f_0_t)] (TDP-R), or an exponential function [exp(-*τ*_0_t)] (TDP-ER), or was modulated in a stochastic manner using a uniform distribution [U(-D%, D%)] (TDP-SR1) or a Poisson distribution [*λ* = 50] (TDP-SR2), all of which were computed *a priori*. In these cases, the amplitude and pulse width of each pulse remained fixed. Figure 2 shows the schematic illustration of the different stimulation sequences.

Stimulation pulse sequences were pre-generated and imported into MCS programming software. A relatively large pool of pulse sequences with a range of parameters were pre-generated. The baseline and modulation parameter settings were guided by the following considerations: (1) The pulse rate and variation follow the neural physiology (e.g., neurons typically do not fire at rates exceeding 400 Hz); (2) The pulse parameters were within device capability; (3) Baseline parameters were set around the default values of the devices (based on the assumption that the trial settings were more likely to be around the device default settings).

The TSP-ETS stimulation was assessed while the trial stimulator was still connected to the implanted leads through OR cable. Afterward, the OR cables were disconnected from the ETS and connected to the MCS stimulator to deliver other test stimulation sequences. Each subject was provided with multiple stimulation sequences, including both static and dynamic stimulation. In this exploratory study, the selection of modulation parameter settings for each study subject was empirical without prior information or optimization. Furthermore, the order of the sequences was applied to a given study subject in a semi-random manner, where: (1) not knowing at the time which sequences might be of most interest, the sequence categories and order presented to a subject were not determined before the study; (2) the selection of stimulation sequences was made such that each sequence category was tested in multiple study subjects; (3) the order of tested sequences was pseudo-randomized with the purpose of minimizing the bias that may be introduced due to the order of test. It should be noted that the number of sequences and categories tested in each study subject varied because the duration of a given study visit varied (patients’ schedules varied and patients could choose to end the test period).

The starting test parameters (e.g., pulse width, rate) for a given subject were guided by: (1) an individual subject's preferred parameters during the standard of care trial, and limited to the ranges of pre-generated stimulation sequence files (pulse widths from 100 to 480 μs, rates from 10 to 200 Hz); (2) an attempt to keep parameters across patients consistent as much as reasonable, to facilitate better cross-subject analysis and comparison. Table 1A below shows the stimulation patterns that were evaluated in each study participant, and Table 1B shows the stimulation parameters that were used for each of the pattern sequences.

**Table 1:** (**A**) Stimulation sequences that were evaluated in each participating study subject are indicated by gray boxes; (**B**) stimulation parameters and the number of times each of the pattern sequences was used (N indicates the number of times that a pulse sequence type was tested). (**A**)

Subject #	TP-ETS	TP-MCS	TDP-Amp	TDP-PW	TDP-R	TDP-ER	TDP-SR1	TDP-SR2
P01								
P02								
P03								
P04								
P05								
P06								
P07								
P08								
P09								
P10								
P11								
P12								
P13								

**Table:** (**B**)

Pulse Sequence Type	Baseline PW (us)					Baseline Rate (Hz)								
	120	200	210	240	480	20	25	40	50	70	80	90	100	200
TSP-ETS (*N* = 10)		7	1	1	1			4	1	2		1	2	
TSP-MCS (*N* = 9)		9						1			2		1	5
TDP-Amp (*N* = 8)		8							6				2	
TDP-PW (*N* = 16)	3			13					8				8	
TDP-R (*N* = 12)		12					4		8					
TDP-SR1 (*N* = 13)		13							9				4	
TDP-SR2 (*N* = 7)		7							7					
TDP-ER (*N* = 7)		7				2			5					

### 2.3 Data collection and analysis

Prior to any stimulation testing and while the external trial stimulator was turned off, a pain drawing, the intensity of overall pain, leg pain and back pain were obtained (based on study subject recall of their pain prior to SCS trial).

For each of the test sequences, stimulation was delivered to the study subject by incrementally increasing the amplitude from 0 mA. The amplitude at which the study subject reported the first definite perception of paresthesia was recorded as “perception threshold”. The amplitude at which the subject felt the stimulation intensity was adequately comfortable and strong enough to do an assessment was recorded as the “testing amplitude”. Study subjects performed the assessment while they experienced stimulation at the testing amplitude. Assessment data for each test configuration included drawings of paresthesia location, ratings of the comfort level of sensation, ratings for helpfulness to pain relief, comparisons to previous stimulation, and open text descriptions of sensations. Verbal descriptions and comments on the stimulation and sensation were also recorded via audio recording if the subject was consented. Subjects were blinded to sequences received while providing assessments of their experience. After the assessment for a given sequence was completed, the amplitude was further increased until the subject reported it as maximally tolerable, and this amplitude was recorded as the “discomfort threshold”.

This initial study was exploratory in nature, and not knowing the nature or magnitude of effects *a priori*, was not powered to achieve statistical significance. Data were analyzed and reported using descriptive statistics to summarize and describe the characteristics of the findings. Descriptive statistics included measure of central tendency (mean or median), measure of variability (minimum, maximum, range, quartiles deviation or variance), and measure of distribution (percentile, frequency distribution or histogram).

## 3 Results

### 3.1 Patient population, demography and characteristics

15 study subjects were recruited for this exploratory study, from which 13 subjects (6 males and 7 females) completed data collection (see Table 2 below). Two subjects dropped out; one cancelled due to an early removal of the trial leads and another did not complete the data collection. Subject average age was 56.5 ± 17.1 years (*N* = 13) and their average time with chronic pain was 11.6 ± 9.8 years (*N* = 13). The average overall, leg only, and back only pain scores prior to the SCS trial (based on recall) were 7.2 ± 1.5, 5.8 ± 2.8, and 7.1 ± 2.3, respectively.

**Table 2:** Demographics of study subjects that completed data collection.

Gender—Females (%)	53.8% (7/13)
Age [Mean (SD)]	56.5 (17.1) years, *n *= 13
Years of Chronic Pain [Mean (SD)]	11.6 (9.8 years), *n *= 13
Baseline pain score [Mean (SD)]	Overall 7.2 (1.5), *n *= 13 Leg 5.8 (2.8), *n *= 13 Low back 7.1 (2.3), *n *= 13

### 3.2 Effect of time-dynamic pulsing on amplitude thresholds

**Figure 3:**
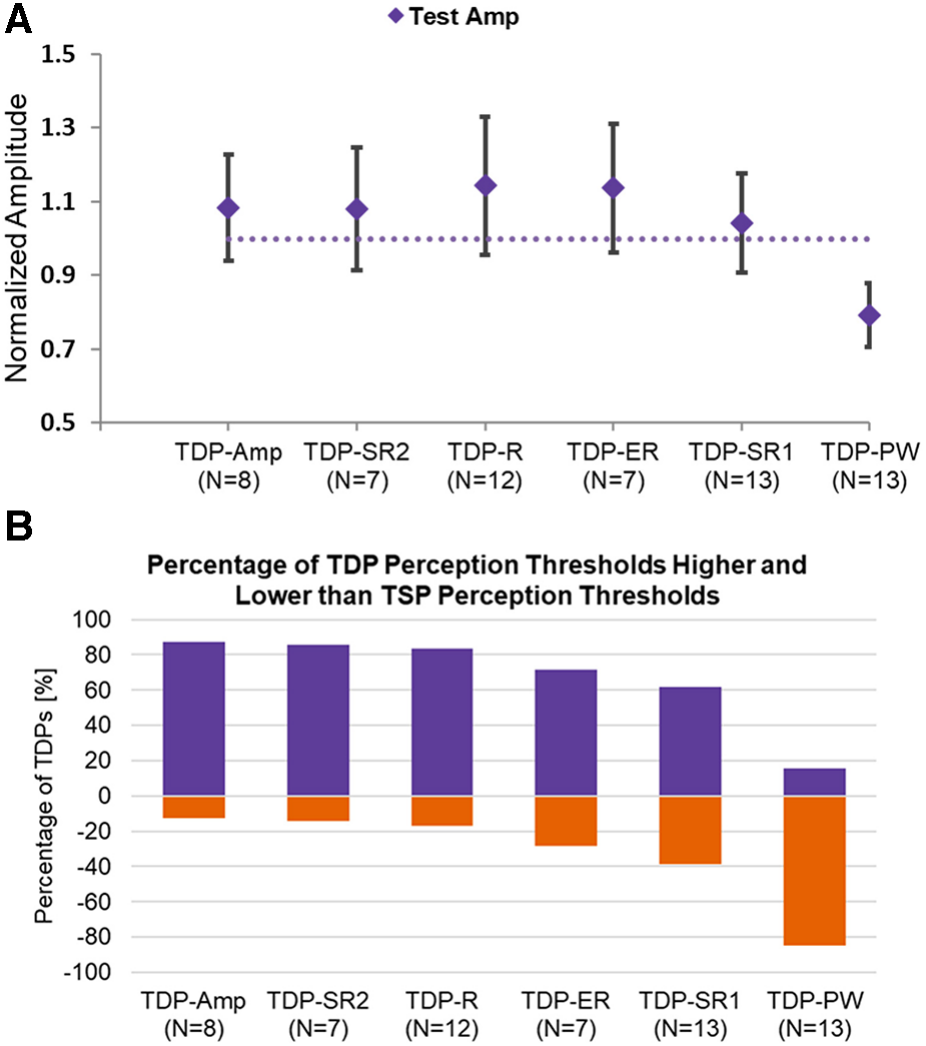
(**A**) Normalized amplitude at test setting for each stimulation type normalized to the corresponding threshold for static stimulation; (**B**) the percentage of testing sets for each stimulation type that had a threshold either higher or equal to (purple/cool shade) or lower (orange/lighter shade) than the static counterpart.

Time-dynamic pulsing schemes tended to modestly elevate amplitude thresholds (perception, testing, and discomfort) as compared to standard time-static pulsing, with the exception of pulse width modulation (TDP-PW). Figure 3A shows the average patient-selected test amplitude for each pattern category, normalized by the test amplitude for the time-static sequence of a given subject. In the case of amplitude modulation (TDP-Amp), the threshold amplitude was the peak amplitude [calculated as Base Amplitude*(1 + D%)], where D represents the modulation depth. As seen in Figure 3A, time-dynamic stimulation using sinusoidal amplitude modulation (TDP-Amp), sinusoidal rate modulation (TDP-R), exponential rate modulation (TDP-ER), and Poisson rate modulation (TDP-SR2) resulted in an increase in the test threshold of about 10 to 20%. Dynamic stimulation using exponential rate modulation (TDP-ER) and uniform rate pulse modulation (TDP-SR1) elevated the perception threshold but not the discomfort threshold (data not shown). The threshold amplitudes for pulse width modulation (TDP-PW) were lower than their static counterpart, presumably because some of the pulse widths in the sequence were larger than the base pulse width (base pulse width was comparable to the pulse width used for static stimulation), and achieved threshold at a lower amplitude. Similar trends were observed in the normalized perception threshold and normalized discomfort threshold, and therefore are not shown here. Figure 3B shows the percentage of testing sets where time-dynamic pulse sequences had perception thresholds either greater or equal to (blue shade) or lower than (orange shade) the perception threshold of the static counterpart, highlighting that for most dynamic modulation sequence types, the threshold is likely to be modestly elevated as compared to the corresponding threshold for static stimulation.

### 3.3 Ratings for stimulation sensation

**Figure 4:**
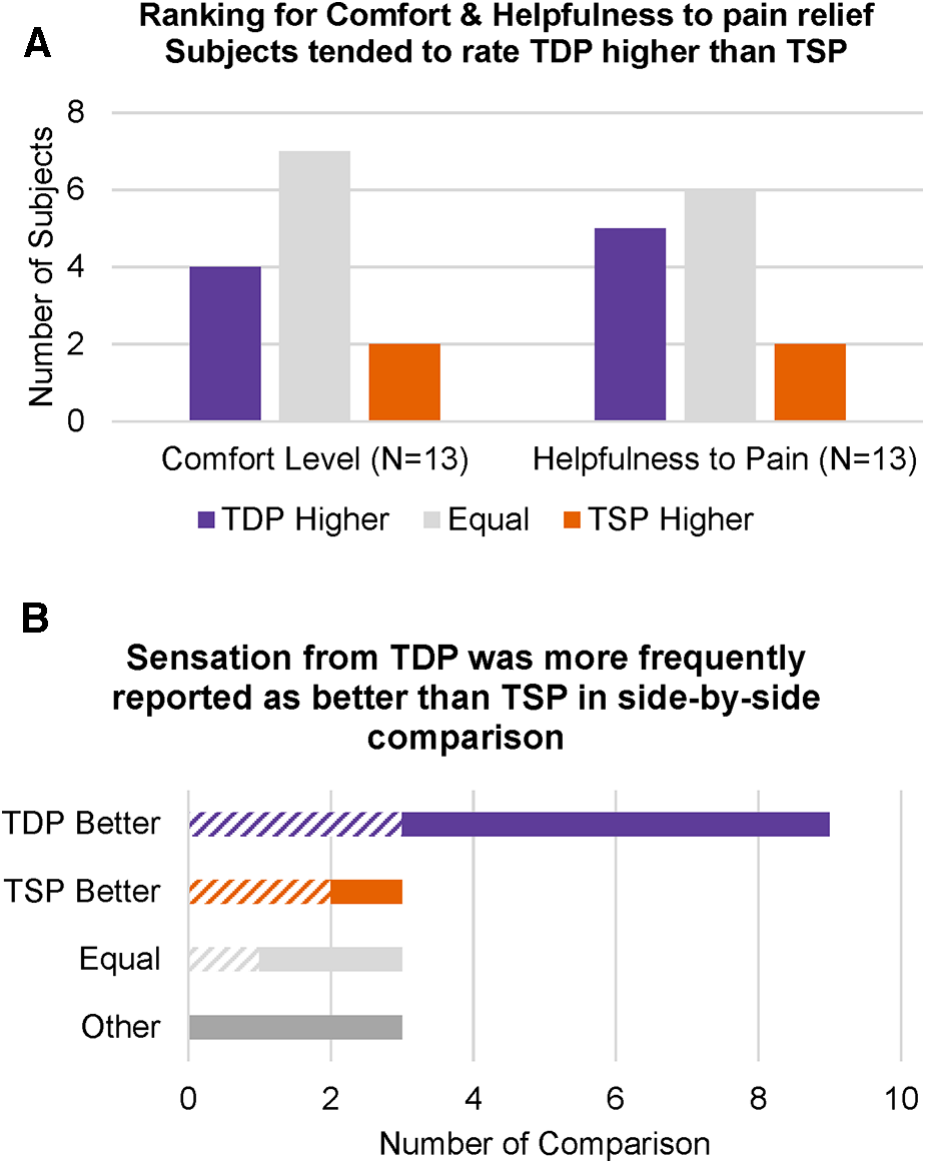
(**A**) Number of subjects whose highest rating for TDP was higher, equal to or lower than their highest rating for TSP, for “comfort level” and “helpfulness to pain relief”, respectively; (**B**) number of side-by-side comparisons where subjects explicitly reported that the TDP or TSP sensation was better, equal to or worse than TSP or TDP, when comparing the current stimulation (TSP or TDP) to the stimulation evaluated immediately before (TDP or TSP). The hatched bars indicate evaluations when TDP was applied before TSP (*N *= 6), and the solid bars indicate evaluations when TSP was applied before TDP (*N *= 12).

During each stimulation, study subjects were asked to complete ratings of (1) comfort level and (2) how helpful they feel the current stimulation will be to their pain relief, and these were presented to them in 5-level Likert scales. For each subject, we compared their highest rating for TDP and their highest rating for TSP. Figure 4A showed the number of subjects whose highest rating for TDP is higher, equal to or lower than their highest rating for TSP, for comfort level and helpfulness to pain relief, respectively. Approximately half of the subjects rated TDP and TSP as equal for both comfort level (7 out of 13) and helpfulness to pain relief (6 out of 13), and among the half of subjects who rated the two differently, more subjects rated TDP higher as compared to the number of subjects who rated TDP lower (*N* = 4 for comfort and *N* = 5 for helpfulness vs. *N* = 2 for both). Subjects were also asked to compare the sensation they were currently experiencing to the stimulation they experienced immediately before, and rate the current stimulation as better, worse, the same or other. Since the order of stimulation types was randomized, this evaluation resulted pairwise comparison of TDP vs. TSP when the TSP was applied before TDP, and comparison of TSP vs. TDP when TDP was applied before TSP. 5 out of 13 study subjects had opportunity to perform comparisons in both directions, with three stimulation sequences transitioned in an order of TDP—TSP—TDP. This assessment resulted in 18 pair-wise comparison between adjacent stimulation, where 10 were obtained from the 5 subjects experiencing two-way comparisons, while 1 was obtained from a TDP—TSP transition, and the other 7 were obtained from TSP—TDP transitions. Figure 4B showed the number of comparison where subjects explicitly reported the sensation from TDP stimulation was better, equal to or worse than that from TSP, when comparing the current stimulation to the stimulation immediately before. The hatched bar shows the evaluation when TDP was applied immediately before TSP (*N* = 6), and the solid bar shows the evaluation when TSP was applied immediately before TDP (*N* = 12). The results showed that sensation from TDP was more frequently rated better than that from TSP (count of 9 vs. count of 3), independent of the order of application. In particular, the 10 pair-wise comparison from the 5 subjects who had opportunities to compare in both directions, showed consistent results in ratings between the two transitions for a given subject in all five cases (i.e., a preference by a given subject for TSP or for TDP was reflected in the comparison in both directions, regardless of testing order). In 3 comparisons, subjects rated the two as the same. In another 3 comparisons, subjects chose the response as “other” and provided comments that they felt the sensations were different. It is likely these subjects had a hard time to determine whether one was better or worse, as opposed to different and equivalent. It is worth noting that no optimization was performed for any dynamic stimulation programmed during the visit, and the static stimulation parameters were chosen to be as similar as possible to the best settings achieved during the trial (which were necessarily static). The ratings showed that without optimization, dynamic stimulation is perceived well by the study subjects.

**Figure 5:**
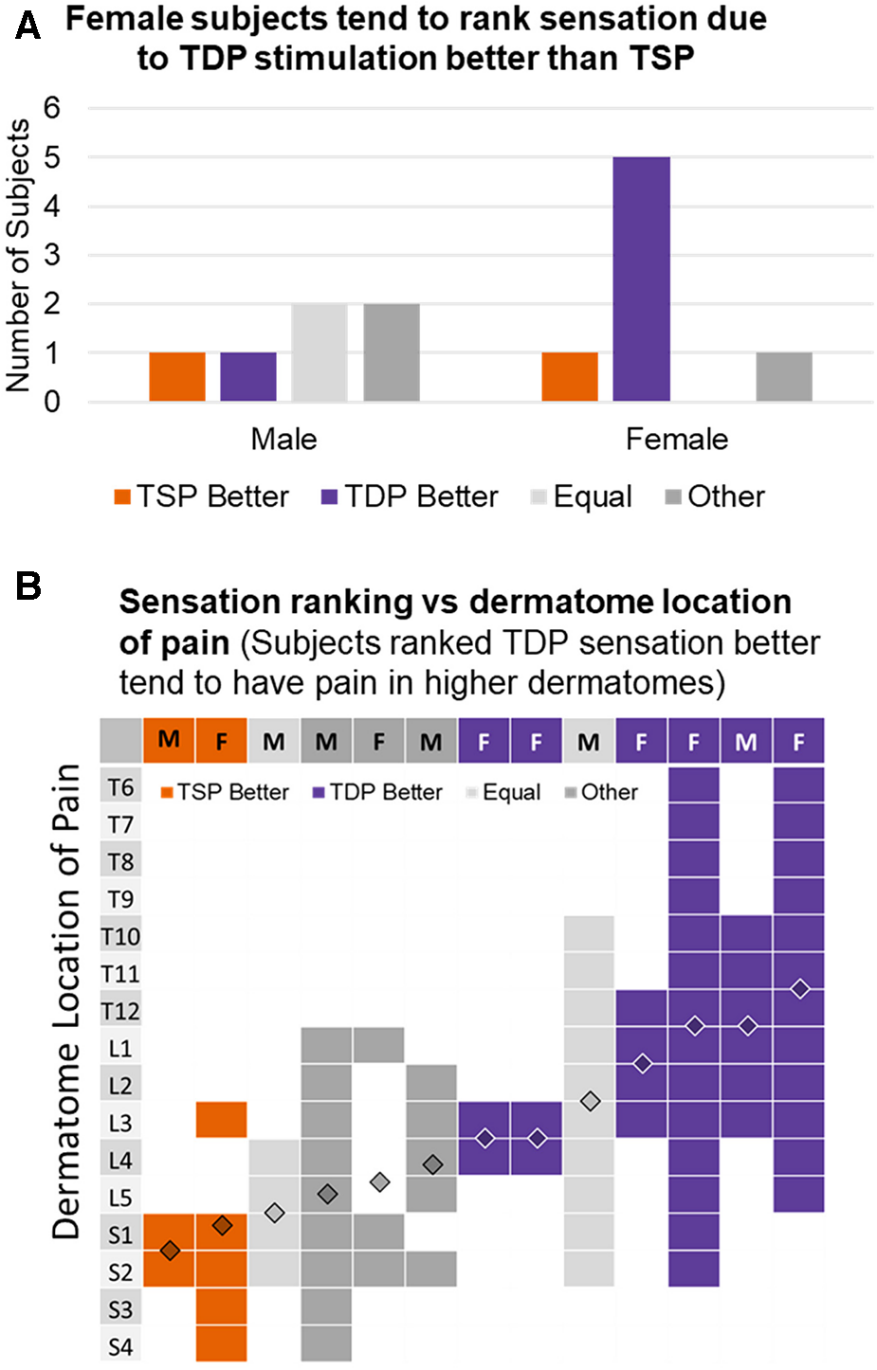
(**A**) Numbers of male and female subjects, respectively, that ranked sensation from TDP stimulation better, equal to or worse than that from TSP simulation; some subjects did not provide clear comparison and selected “Other” with a note of feeling differently; (**B**) discret dermatome distribution determined from subjects’ drawings of pain with subject labels color-coded to indicate their ranking of sensation in favor of TDP, TSP, equal, or other.

A more careful examination of the population that rated in favor of TDP over TSP revealed differences by subjects' gender and pain location. Figure 5A showed the number of male and female subjects respectively, that ranked sensation from TDP stimulation better (purple), equal to (light gray) or worse (orange) than that from TSP simulation. Some subjects did not provide clear comparison and selected “Other” with a note of feeling only a difference (dark gray). Among the six male subjects, there is no clear trend observed for ranking preference, while among the seven female subjects, the majority (5 out of 7) ranked the sensation from TDP stimulation as better. The subject's ranking preference also appeared to be related to the subject's location of pain. Figure 5B shows a map of the discrete vertebral dermatome distribution as determined from the subjects’ drawings of pain, with each column representing a subject and each row representing a dermatome. Each subject (column wise) was color-coded to indicate their ranking of sensation comparing TDP vs. TSP as TDP ranked better (purple), TSP ranked better (orange), equal (light gray) or other (dark gray). The filled vs. blank cell entries of each column represent the binary coding of the overlap of a subject's pain drawing with a reference dermatome map (https://i.pinimg.com/736x/ef/76/47/ef7647ceae98d10588f14b4ecd7e6a89.jpg). If the pain area overlapped at least partially with a dermatome area covered by a spinal nerve (S4—S1, L5—L1, T12—T6), the corresponding dermatome is binary coded as 1 with corresponding cell entry filled in solid color; otherwise, it is coded as 0 with the corresponding cell entry left blank. The overlaid diamond graph shows the estimated centroid of the dermatome level averaged from the binary representation. Subjects with pain at higher dermatomes tended to rank sensations from TDP stimulation as better, while subjects with pain at lower dermatomes tended to rank sensation from TSP stimulation as better. Subjects with pain at intermediate dermatome level tended to rate them as equal or other.

### 3.4 Paresthesia coverage

**Figure 6:**
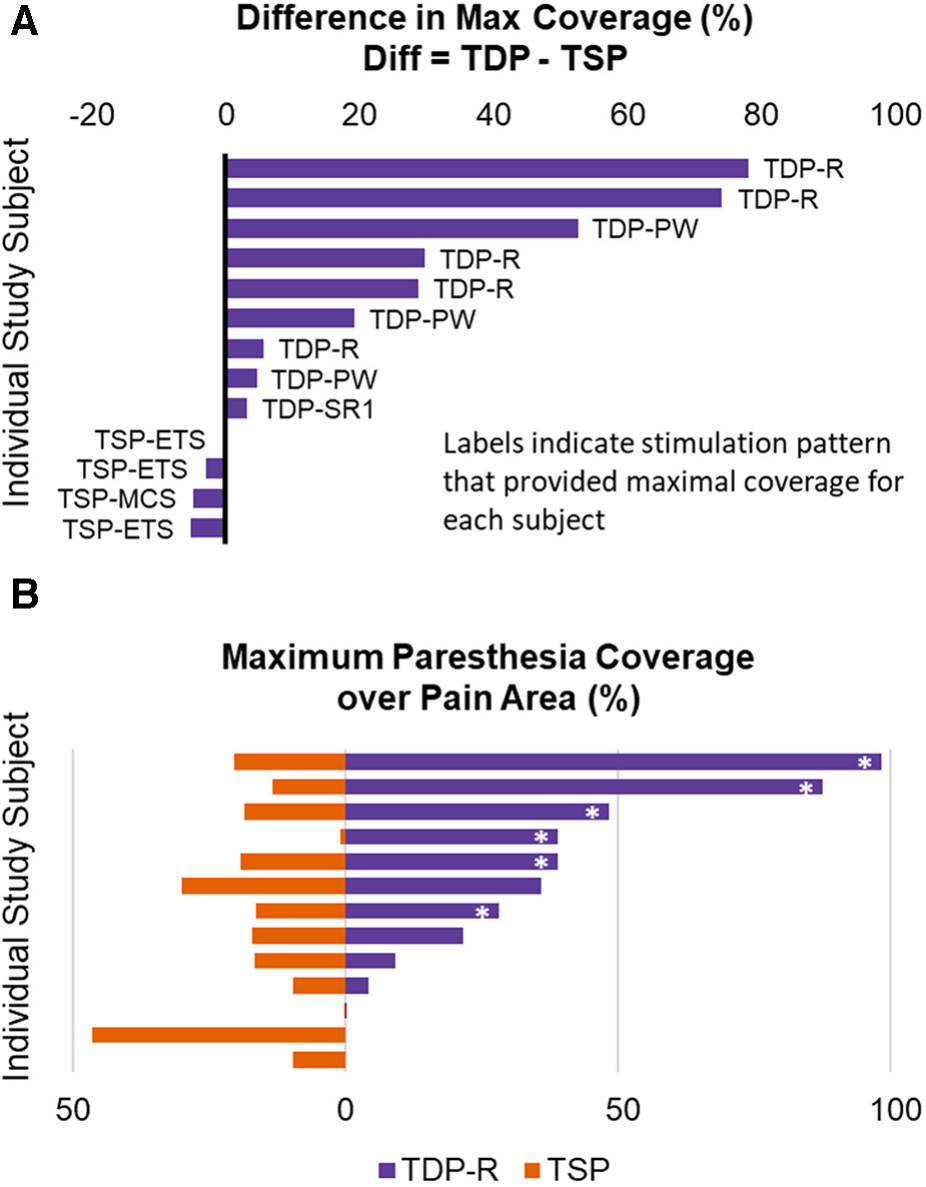
(**A**) Difference in maximal percent pain coverage achieved in each subject by a TDP vs. by a TSP stimulation type, annotated with the specific stimulation type that achieved the maximum in each subject. (**B**) Pair-wise comparison of the maximal percent pain coverage achieved in each subject by TDP-R vs by TSP stimulation. The asterisk * marks the cases where the coverage by TDP-R was at least 10% greater than TSP coverage, to distinguish from cases where the differences were small.

The percent of pain coverage by paresthesia produced by each TDP stimulation, was compared to that achieved by TSP stimulation, as shown in Figure 6. Figure 6A shows the difference in maximal percent pain coverage achieved in each subject by TDP vs. by TSP stimulation, annotated with the corresponding pulse type that achieved the maximum coverage. For each subject, the difference was calculated as maximal percent coverage achieved by any TDP stimulation minus the maximal percent coverage achieved by any TSP stimulation. Results show that maximal coverage was achieved with at least one dynamic sequence in 70% (9 out of 13) of subjects. In most cases where TDP stimulation achieved better coverage, the difference was greater than 20% (5 out of 9), while in all cases where TSP stimulation achieved better coverage, the difference was less than 10%. It should be noted that the data also suggested that sinusoidal rate modulation (TDP-R) may have the highest potential (of those tested) to achieve greatest pain coverage; it produced the maximal coverage in 5 out of 9 subjects for whom TDP stimulation provided better coverage over the pain area. To further evaluate the potential of TDP-R for producing greater pain coverage, a pair-wise comparison was performed between the maximal percent pain coverage achieved in each subject by TDP-R vs. by TSP stimulation (see Figure 6B). In 8 out of 13 subjects, the maximal coverage by the TDP-R was larger than its TSP counterpart. In 6 out of 13 subjects, the coverage by TDP-R was at least 10% greater than TSP coverage. A paired t-test (one-way) indicated that greater pain coverage by TDP-R stimulation vs. that by TSP stimulation was statistically significant (*p *= 0.016). Similar analysis was done comparing maximum pain coverage for all categories of rate modulated stimulation vs. static stimulation (greater coverage with rate modulated stimulation, *p *= 0.015), and with all categories of dynamic stimulation vs. static (greater coverage with all dynamic stimulation, *p *= 0.011). Results from these evaluations suggested that dynamic stimulation tends to achieve greater paresthesia coverage. It should be noted that all the test stimulations used the same electrode configuration, comparable baseline settings for pulse width and rate, and the user-preferred stimulation intensity for a given test waveform.

### 3.5 Description of paresthesia sensation

**Figure 7:**
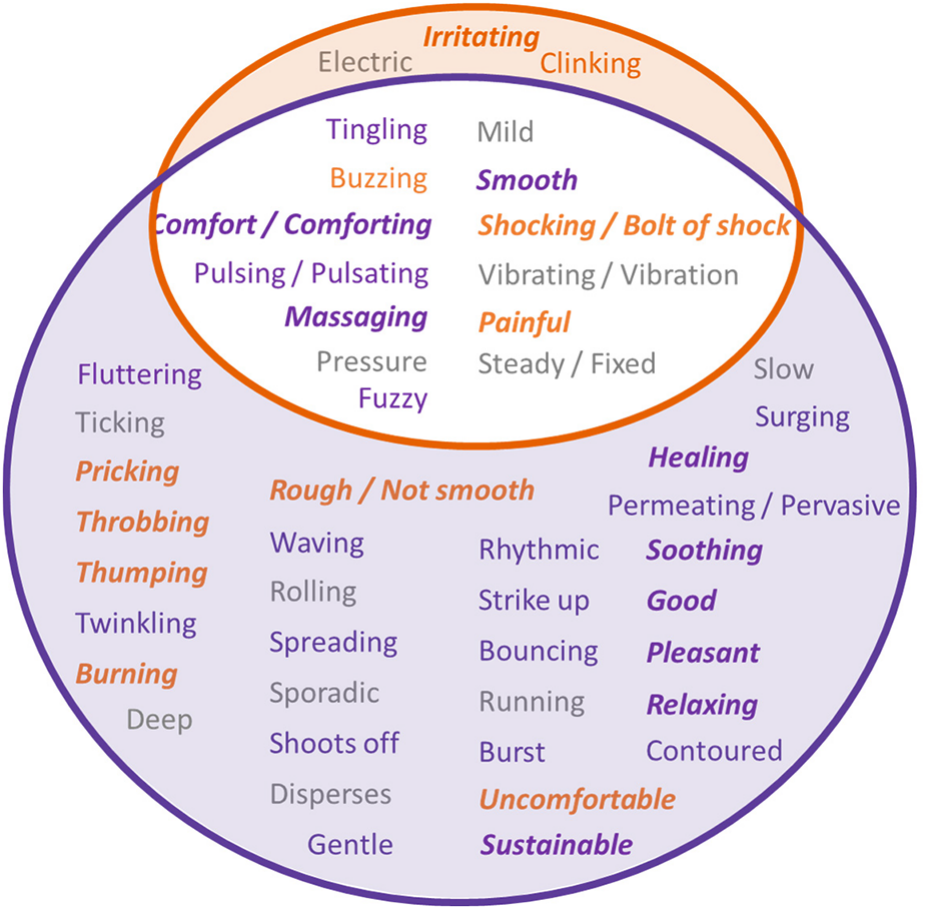
Descriptive phrases/words that the study subjects used to explain their sensations for each stimulation type, with description for TSP circled in orange, and description for TDP circled in purple. The intersection are the common phrases/words that have been used for both types of stimulation. The color/font combination of the phrases/words indicate their likely connotation assessed using ChatGPT, as Negative (bold orange italic), Slightly Negative (orange), Neutral (gray), Slightly Positive (purple), or Positive (bold purple italic).

A description of words used to describe the sensations produced during stimulation was collected via a written questionnaire and verbally via an audio recording. The form of the data was an open response to a question, and the study subjects could choose whatever language they felt best described the sensation. Patients were always blinded to the type of stimulation they were receiving. Descriptive words/phrases that the study subjects used to explain sensations were either extracted from their written response or transcribed from the audio recording by an independent reviewer that has not been involved in the data collection. The descriptive words/phrases for each stimulation type were noted in a Venn diagram in Figure 7, with descriptive words/phrases used for TSP circled in orange, and descriptive words/phrases for TDP circled in purple. The words/phrases were also categorized by their likely connotation into five groups using a deep learning language model (ChatGPT developed by OpenAI, based on the GPT-3.5 architecture, with a knowledge cutoff in January 2022) each represented with color/font combination as either Negative (bold orange italic), Slightly Negative (orange), Neutral (gray), Slightly Positive (purple), or Positive (bold purple italic). The intersection shows words/phrases that were used for both types of stimulation in at least one instance. 13 of 48 (27.1%) words/phrases were used to describe both TSP and TDP stimulation types, 3 of 48 (6.2%) were used only in connection with TSP stimulation, and 32 of 48 (66.6%) were used only in connection with TDP stimulation, including numerous additional words categorized as Positive or Slightly Positive. For each type of stimulation (TDP or TSP), the words/phrases showed a balanced distribution across the five scales of negative/positive connotation.

6 out of 13 subjects mentioned in the audio recording that their experience was better than the trial and 2 subjects expressed their desire for having some of the dynamic SCS settings during their trial. Some subjects also expressed their desire to have options and would like to be able to use different settings under different situations, such as, daily vs. night use, use during periods of relaxation vs. actively moving, or use only during episodes when their pain is particularly high.

## 4 Discussion

Spinal cord stimulation has been successfully used for the treatment of chronic neuropathic pain, with stimulation intensity set either above or below patients’ perception, providing paresthesia-based or paresthesia-free therapy, respectively. Response and preference to the two SCS therapy modalities varies across patients, showing both are important therapeutic options (18). There have recently been many efforts to advance sub-perception stimulation therapy. One group has recently focused on improving the amplitude control of paresthesia-based stimulation and showed that there is some value (38), but there have been relatively fewer efforts to improve paresthesia-based stimulation therapy. Here, we report the results of an exploratory study to determine if there is observable signal using SCS with dynamic pulse trains to enable tailoring of the paresthesia sensation, improve the coverage, and improve the opportunity for pain relief. To our knowledge this is the first clinical study that has evaluated the effect of multiple categories of temporally modulated stimulation pulse trains on induced sensation in patients receiving SCS trials, including amplitude modulation, pulse width modulation and rate modulation using different modulation functions. Tan et al. (34) evaluated the effect of intensity modulated stimulation (IMS) on the clinical effect of SCS, as an alternative to conventional tonic stimulation. In their study, only pulse width modulated stimulation was evaluated, which, consistent with the results of the present study, was shown to produce more comfortable paresthesia sensation. A similar degree of pain relief during SCS trials was reported in that study, whether using pulse width modulated or tonic stimulation. The evaluation in present study included pulse width modulation and the modulation of the other two major pulse parameters, amplitude and rate (sinusoidal, uniform, and Poisson-modulated), for the effects during SCS trial.

It is important to understand the limitations of this study. First, this was an exploratory study, with a total of 15 subjects enrolled of which 13 completed the study visit. Second, the assessments were conducted acutely in the clinic, during study sessions of up to two hours for each subject. Subjects were able to decide when they would like to stop the data collection, resulting in variation in the number of tests and types of pattern categories that could be tested in each subject. Third, despite effort to reduce cross subject variation, there was still variation in stimulation parameters applied to each individual subject, thus in most analyses the sample size may not be large enough to support statistical analysis. Data were analyzed and reported using descriptive statistics to summarize and describe the characteristics of the findings. Fourth, each stimulation pattern that the study subjects experienced was brief, running only for a few minutes, so patient feedback was likely their first or immediate response, and long term effects of time-dynamic SCS could not be assessed. And last, due to the study time limitations, we could not optimize paresthesia coverage or other parameters, as is normally done in SCS related studies.

Subjects in general perceive the dynamic stimulation as comfort and helpful to their pain relief. When comparing sensation from the two types of stimulation, more subjects reported the sensation due to dynamic stimulation as better or at least equal to that due to static stimulation. The gender-specific difference observed in sensation ranking, with female subjects tending to rank sensation from dynamic stimulation better than that from static stimulation, is very interesting, despite the small sample size. Gender-related impact on pain and response to pain treatment has become a topic of scientific and clinical interest in recent years (39), including chronic pain and SCS treatment (40–42). Although observations from different retrospective studies seems to vary, with gender-based difference in SCS efficacy observed in some studies (40) but not in other studies (41, 42), there is still considerable interest in gender-dependent efficacy, as recent discoveries have suggested there are gender-specific endogenous pain pathways (39).

The relationship observed between the pain location and the ranking for dynamic vs. static stimulation is also of particular interest. Subjects with extremity pain seemed to prefer static stimulation, while subjects with axial back pain seemed to favor dynamic stimulation better. More in depth investigation is required to confirm the presence of such a relationship. While it is not be conclusive due to the small sample size and acute nature of the study, this finding suggests it is possible that paresthesia based SCS treatment can be optimized for pain at different body locations using stimulation patterns.

Of note, Figure 5B shows that most female patients in our cohort had back pain while most male patients in our cohort had lower extremity pain, making it difficult to determine whether gender or dermatomal location of pain might be the primary factor in the apparent preference (or not) for time-dynamic stimulation. Additional research is needed to understand this possibility better.

Results from Figure 6 showed that stimulation using time-dynamic pulses has the potential for expanded therapy coverage, suggested by the increased paresthesia coverage when using dynamic stimulation vs. static stimulation with the same electrode configuration, comparable pulse width, and without optimization of any parameter. Some subjects described the paresthesia coverage under time-dynamic stimulation as “spreading”, “permeating” or “pervasive” (Figure 7), suggesting a dynamic expansion of paresthesia sensation. Tan et al. observed that using intensity-modulated SCS produced a perception of radial or linear radiation (34). SCS induced sensation of paresthesia is caused by the orthodromic activation of afferent fibers. The location of paresthesia is determined by the population of afferent fibers that were activated and the corresponding topographic map or receptive field that these fibers innervate. Both the amplitude and pulse width modulation can be categorized as charge intensity modulation. One possibility for the observation of expanded coverage using time-dynamic pulses is that different nerve fiber populations may have been recruited by pulses of different charge intensity in an asynchronous manner (33). For example, lower threshold fiber population may be recruited at smaller effective pulse interval and higher threshold fiber population may be recruited at a larger effective interval. The increased variation in recruited population and the dynamic variation in their activation rate may be associated with the different paresthesia coverage as compared to the static coverage. In the rate modulated stimulation, it may be less likely that different fiber population was recruited, as the pulse width and amplitude for each pulse were constant and comparable to those same parameters for the static pulses. The changes in paresthesia coverage associated with rate modulated stimulation as compared to those associated with static stimulation may be a result of difference in secondary or higher order downstream neural processing activities that code perception, given that the activation and output of the downstream neurons along the transmission pathway may differ depending on the temporal patterns of the input spike (action potential) train. Studies have shown that temporal summation of excitation and inhibition at the synapse can establish diverse responses to different rates of presynaptic input (43, 44).

Some subjects described their paresthesia under time-dynamic stimulation (especially rate modulated stimulation) as “deep massaging” and reported feeling the stimulation “reaching down to where the pain is”, or “going across the pain area, rather than just being at the surface”. An explanation to the difference in perceived depth of paresthesia coverage achieved with dynamic vs. static SCS may also be attributed to the difference in secondary or higher order downstream neural activities that produce the perception of expanded coverage. It could also be associated with the dynamic variation in the intensity of sensation induced by the rate modulated stimulation, which may result in a radiating sensation. Some subjects did describe the sensation as “waving/rolling” or “permeating/pervasive” (see Figure 7). Another explanation might be that receptive fields (RFs) and sensory maps may not be simply attributed to individual neurons (RFs) or population of neurons (maps), but may also depend on interactions between neurons (45). That is, the difference in location of perceived paresthesia under dynamic and static stimulation may be a result of different patterns of neural activity in a collection of central neurons and the difference in integration along higher order pathways.

Human sensory perception involves complex neuronal activity, including the activation of multiple neuronal populations with varying properties and functions (45–48). The recruitment of nerve fiber populations of different size under intensity (amplitude and pulse width) modulated stimulation may result in not only a different sensation location, but also a different quality of sensation. Neurophysiologist believes that it is the responses from multiple populations of mechanoreceptive afferents rather than individual afferents that mediate the perception of stimulus intensity (45, 48). Different fiber can convey information about different aspects of the stimulus which are integrated to form the percept of stimulus (47). In addition, the asynchronized fiber recruitment pattern may be more similar to natural neural firing patterns when stimulus induced action potentials propagate from peripheral to spinal cord at delay due to variation in timing of activation and transmission velocity.

It is also believed that the time series of action potentials (spike trains) is more important in information transmission. In both peripheral and central neurons, the generation of series of action potentials or spike trains is a dynamic and time-varying procedure (49, 50), which is more complex than single frequency tonic pulse trains that are typically delivered from neurostimulators. Sensory perception is the central processing of sensory stimuli into a meaningful pattern, which is essentially a process involving pattern recognition. The neuronal firing patterns encode the information about the external stimulation and the brain decodes the patterns for perception (51, 52). Paresthesia induced during conventional SCS using tonic stimulation pulses trains is often described as “tingling”, “steady” or “fixed” which may be unnatural. In this study, although tingling was still the most frequently used word to describe the sensation for both dynamic and static stimulation, for which part of the reasons is that the study subjects, as an SCS trial patient, may have been exposed to this word during their routine procedure education where “tingling” may have been most commonly used to describe the sensation that they would expect to feel during the trial for paresthesia-based SCS therapy. The dynamic stimulations, especially the sinusoidal rate modulated stimulations, were commented by some study subjects as more natural or more contoured to their body. The sinusoidally modulated rate of stimulation was designed to resemble the firing patterns of Slowly Adapting Type II (SA II) afferents (such as those transmitting response from Ruffini endings) in response to constant or slowly changing stimuli, with firing rates increasing or decreasing with intensities of stimuli (53). The exponentially modulated rate of stimulation, was designed to represent the firing pattern of slowly adapting type I (SA I) afferents (such as Merkel discs) in response to ramp-and-hold stimuli, which exhibits an exponential decrease in the firing rate during the hold phase (53). The exponentially decaying firing rate did not seem to be favored by study subjects, though some subjects still thought it could be helpful for pain relief. This is probably because the higher firing rate of SA I afferent at the beginning of stimulus is supposed to signal the onset of stimulus and inducing an alerting response. Patients may not welcome this alerting sensation that may keep reminding them something is going on. In future studies, it may be of interest to evaluate stimulations with rates modulated with an increasing exponential function and see if it will have potential to produce sensation similar to that induced by sinusoidal rate modulated stimulation. Overall the comments regarding more or less natural sensation provided by study subjects suggested that designing stimulation pulses that are more neural-mimetic may provide additional way to improving the paresthesia-based SCS therapy, thus further increasing the clinical use of SCS for pain control.

Paresthesia induced by static stimulation were also more often described as “steady” or “fixed”. On contrast, paresthesia induced during the dynamic stimulation were more likely being described as dynamic including pulsing, waving, bursting, surging and such, which also inspired the naming of the time-variant stimulation sequences. There was more variation in the languages that the subjects used to describe the sensations they experienced during dynamic stimulation as compared to what they used to describe static stimulation. Some sensations were distinct and unique and were only perceived during dynamic stimulation. This suggested that stimulation using time-dynamic pulses may produce manifold paresthesia sensations that patients may perceive during paresthesia SCS therapy. Some study subjects commented that the sensation under dynamic stimulation felt more natural or more tolerable, as compared to static stimulation. This may be attributed to the fact that nerve system transmits information about sensation in dynamic spiking patterns instead of tonic patterns. The positive comments and preference by study subjects as they compared the testing stimulation with their trial stimulation, as well as the desire that some subjects expressed to have the testing stimulation during their trial, suggested that SCS using dynamic stimulation can be a promising paradigm to improve the patients’ overall experience with this type of therapy. Many study subjects also expressed their desire for having multiple options or preference to different stimulation during different activities, suggesting it is important to tailor the stimulation induced sensation to each subject's preference.

Due to its acute nature, the study focused on evaluating the quality of sensation that study subjects experienced during spinal cord stimulation. The stimulation was not long enough for a reasonable evaluation of pain relief, which is the primary therapeutic objective of spinal cord stimulation. The subjects' response to the questionnaire regarding rating the helpfulness to pain relief showed that they perceive both dynamic and static stimulation as helpful or very helpful to relieving their pain, although some may rate dynamic stimulation higher over the static stimulation, or vice versa. Some subjects also expressed their desire for using different stimulation under different pain condition or physical activities. It suggested that the dynamic stimulation may provide additional options for customizing SCS to meet variant patient needs. In a rodent model of chronic constriction injury, Edhi et al. (37) demonstrated that most time-dynamic pulse (TDP) stimulation similar to what has been tested in this study significantly reversed mechanical hypersensitivity, except for pulse width modulated stimulation. The anti-nociceptive effects of some TDP stimulations even outlasted SCS duration, suggesting that TDP modulation may produce therapeutic pain-relieving effect and possibly improving clinical outcomes by improving the sensory experience. Additional studies are warranted to evaluate the efficacy of dynamic stimulation on pain relief and patients' satisfaction in chronic settings.

In this study, all dynamic and static stimulations were applied at a supra-perception level, in order to test the hypothesis that dynamic stimulation may provide additional options to improve paresthesia sensation and coverage. Observations of responses from study subjects suggested that the dynamic stimulation were well perceived by study subjects under supra-perceptive or paresthesia based SCS. Although not tested in this study settings, it could be hypothesized that sub-perceptive or non-paresthesia SCS could also benefit from using such more physiologically relevant stimulation patterns, likely with similar underlying neural mechanism. Evaluating effect of dynamic stimulation for sub-perceptive SCS may require longer duration of stimulation and may be evaluated in future studies with longer follow up.

Another challenge in SCS therapy is the loss of effectiveness over time for some patients (54, 55). It is sometimes described as build-up of “tolerance” to the therapy, a phenomenon of diminished response to treatment observed in pharmaceutical and medical interventions when repeated treatment is presented. Patients may have to increase the stimulation intensity to achieve the needed analgesia or efficacy was lost at all. Although the loss of efficacy could be attributed to a variety of reasons, such as physiological changes or technical complications (56), neural adaptation to constant stimulation could be one of the important factors (57, 58). Alternative pulse trains to repeat tonic stimulation, for example, bursting stimulation, may partially address this challenge by cycling between On and Off stimulation periods. However, the intra-burst and the inter-burst parameters still remain constant over time (59, 60). Technologies incorporating dynamic variations in stimulation parameters such as the ones evaluated in this study may have a greater potential of reducing or delaying the build-up of tolerance or neural adaptation, potentially leading to therapeutic longevity.

In summary, our result suggests that dynamic SCS may enable tailoring of the paresthesia sensation and coverage that patients perceived during stimulation, potentially improving patients' pain-relieving experience. The results suggest that personalizing and providing access to multiple dynamic stimulations may hold clinical value and potential to improve the outcomes of SCS for the treatment chronic pain.

## Data Availability

The manuscript reports original clinical trial data of NCT02988713. Data for this study can be made available to other researchers upon request in compliance with security and privacy regulations and in accordance with Boston Scientific's data sharing policy (https://www.bostonscientific.com).
